# Experimental data from service-like creep-fatigue experiments on grade P92 steel

**DOI:** 10.1016/j.dib.2023.109333

**Published:** 2023-06-22

**Authors:** Nadja Sonntag, Maria Jürgens, Patrick Uhlemann, Birgit Skrotzki, Jürgen Olbricht

**Affiliations:** Bundesanstalt für Materialforschung und -prüfung (BAM), Division 5.2 Metallic High Temperature Materials, 12205 Berlin, Germany

**Keywords:** Tempered martensite-ferritic steel, P92, Creep, Stress relaxation, Creep-fatigue, Dwell times, Hold times, High temperature

## Abstract

This article refers to the research article entitled “*Creep-Fatigue of P92 in Service-Like Tests with Combined Stress- and Strain-Controlled Dwell Times*” [1]. It presents experimental mechanical data from complex service-like creep-fatigue experiments performed isothermally at 620°C and a low strain amplitude of 0.2 % on tempered martensite-ferritic grade P92 steel. The datasets in text file format provide cyclic deformation (minimum and maximum stresses) and the total (hysteresis) data of all recorded fatigue cycles for three different creep-fatigue experiments: 1) a standard relaxation fatigue (RF) test with symmetrical dwell times of three minutes introduced at minimum and maximum strain, 2) a fully strain-controlled service-like relaxation (SLR) test combining these three-minute peak strain dwells with a 30-minute dwell in between at zero strain, and 3) a partly stress-controlled service-like creep (SLC) test combining the three-minute peak strain dwells with 30-minute dwells at constant stress. Such service-like (SL) tests with additional long-term stress- and strain-controlled dwell times are non-standard, rare, and expensive, making these data very valuable. They may be used to approximate cyclic softening in the technically relevant range, for the design of complex SL experiments, or for detailed analyses of stress-strain hystereses (e.g., for stress or strain partitioning methods, for the determination of hysteresis energies (work), inelastic strain components, etc.). In addition, the latter analyses may supply important input for advanced parametric lifetime modeling of components under creep-fatigue loading or model calibration parameters.

**Specifications Table**

**Table:** 

Subject	Materials Science; Engineering
Specific subject area	High-Temperature Creep-Fatigue Testing
Type of data	Tables in *.lis format These files contain all relevant data as tab-separated ASCII. The files may directly be opened using most text editor/notepad apps. Similarly, the contents can directly be imported to any spreadsheet software (e.g. MS Excel).
How the data were acquired	Fatigue Tests: Three types of complex creep-fatigue experiments were conducted isothermally at 620°C in air on cylindrical, grade P92 steel specimens with uniform cross-sections and ground surfaces: 1) strain-controlled relaxation fatigue (RF) test with strain-controlled three-minute (180 s) dwell (hold) times at minimum and maximum strain in each cycle; 2) service-like relaxation fatigue (SLR) with strain-controlled three-minute dwell times at minimum and maximum strain and 30-minute (1800 s) dwells at constant strain (ε =0) and 3) service-like creep-fatigue (SLC) with strain-controlled three-minute dwells at minimum and maximum strain and 30-minute dwells at constant stress (σ (ε =0)). Equipment: Servo-hydraulic testing machine (MTS Landmark, MTS, Eden Prairie, USA); water-cooled high-temperature extensometer (MTS-632.51F.04, MTS, Eden Prairie, USA); inductive specimen heating; type S thermocouple, spot-welded to the center of the gauge length of specimens; plug-in high stability temperature controller (Eurotherm 2604, Schneider Electric Systems, Limburg, Germany) Data Acquisition: Raw data (time, *t*; number of cycles, *N*; force, *F*; extensometer strain, *ε*; temperature, *T*) were acquired by MTS MPT (MultiPurpose TestWare) software. For the analyzed raw data, forces were replaced by stresses to enable geometry-independent access to the material behavior.
Data format	Analyzed Raw
Description of data collection	This collection contains mechanical data of three different complex high-temperature dwell-fatigue experiments. Each data file consists of three parts: 1) meta-data of experimental conditions, 2) extracted peak stresses, peak strains, and corresponding control temperatures for each analyzed fatigue cycle (*N*), and 3) time-, stress-, strain-, and temperature data of complete fatigue cycles.
Data source location	Bundesanstalt für Materialforschung und -prüfung (BAM), Division 5.2 Berlin Germany
Data accessibility	The data is publicly available online at: Repository name: Zenodo Data identification number: https://doi.org/10.5281/zenodo.7198218 Direct URL to data: https://zenodo.org/record/7198219#.Y0lGitfP1aR
Related research article	N. Sonntag, M. Jürgens, B. Skrotzki, J. Olbricht, Service-Like Creep-Fatigue Testing with Combined Stress- and Strain-Controlled Dwell Times, Int. J. Fatigue 168 (2023), 107381. https://doi.org/10.1016/j.ijfatigue.2022.107381

## Value of the Data

• The data in this article relates to complex (non-standard) creep-fatigue experiments with combined stress- and strain-controlled dwell times and extraordinarily long dwells that make these tests very expensive and valuable.
• They provide insight into the fundamentally different mechanical behavior during long-term stress- and strain-controlled dwell times in creep-fatigue experiments and demonstrate their effect on subsequent transient load shifts and dwell times.
• Because the high-temperature tests were performed at a low mechanical strain amplitude (in the technically relevant elastic-plastic deformation range), the data are of interest to both materials science and industry (e.g., the nuclear or thermal power plant industry). The current experiments were performed on one widely used steel grade for steam pipelines. It is anticipated that the demonstrated impact of the different load profiles on the deformation and lifetime is qualitatively similar for the whole material class, i.e., all 9-12% chromium tempered martensite-ferritic steels.
• The data can be used to approximate cyclic softening in the technically relevant range, for the design of complex SL experiments, or for detailed analyses of stress-strain hystereses (e.g., for stress or strain partitioning methods, for the determination of hysteresis energies (work), inelastic strain components, etc.). In addition, the latter analyses can supply important input for advanced parametric lifetime modeling of components under creep-fatigue loading or model calibration parameters.

## 1 Objective

The objective of this article is two-fold: First, it highlights the relevance of the data for different interest groups, which is based on the special, non-standard loading paths that were applied in the related mechanical experiments (cf. previous section for further details). Second, it provides additional details on the test setup, data structure and applied analysis methods (cf. following section). Thereby, it guides possible users of the datasets through the provided files and ensures proper analysis/interpretation of the available data. This exceeds/extends the scope of the original research article that was limited to a description and phenomenological interpretation of the observed material behavior.

## 2 Data Description

One summarizing *.lis file is provided for each of the three dwell-fatigue experiments, namely relaxation fatigue (RF), service-like relaxation fatigue (SLR), and service-like creep-fatigue tests (SLC), all of which are indicated in the file names. The file format *.lis is SQR output file format (tab-delimited) that can be opened like ASCII *.txt or *.dat files by e.g., text editors, MS Excel, or common data analysis software. Each data file consists of three parts in a broad-to-specific order, as described in detail in the following:

### 2.1 File name and meta-data of experimental conditions (headers)

This first file section summarizes general information on the testing parameters/conditions, machine, and specimen geometry. The number of cycles to failure ($N_{f}$) and the total number of fatigue cycles (N) are also indicated in this upper file part.

### 2.2 Max-min data

**Fig. 1:**
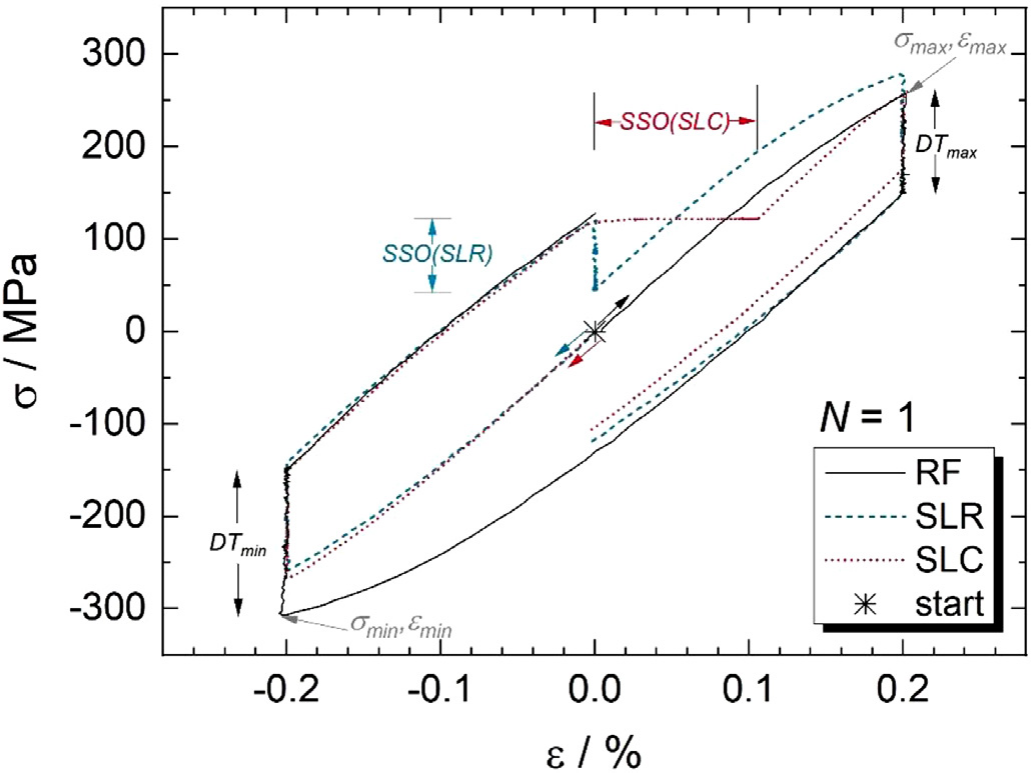
Stress-strain hystereses for the first cycles (N=1) of relaxation fatigue (RF), service-like relaxation (SLR), and service-like creep (SLC) tests. Colored arrows indicate the loading sequences next to the hysteresis start point (asterisk). Positions of peak stresses ($\sigma_{min}$, $\sigma_{max}$) and strains ($\varepsilon_{min}$, $\varepsilon_{max}$) in an individual hysteresis are exemplarily indicated in gray for the RF test. Adopted from [1].

This section starts with the sub-heading/entry “[MAX-MIN]”. The peak (maximum and minimum) values of stress, strain, and temperature are listed for all analyzed fatigue cycles (“Cycle No”) in this section. They were extracted from the total data given in the third file section and are marked for $\sigma_{min}$, $\sigma_{max}$ and $\varepsilon_{min}$, $\varepsilon_{max}$ in the stress-strain hystereses of the first cycles in Fig. 1 exemplarily. The provided mechanical stresses (*σ*) were determined from mechanical forces (*F*) and the initial cross-section (*A_0_*) of the samples at room temperature via σ = *F* / *A_0_*.

### 2.3 Total data of individual fatigue cycles

This section starts with the sub-heading/entry “[Data]”. Time-dependent stress-, strain-, and temperature data are provided in this final file section for a reduced number of complete fatigue cycles, as standard in fatigue data analysis.

The following cycles were analyzed:

• all cycles for 1 < *N* ≤ 10,
• every 10^th^ cycle for *N* > 10,
• and, additionally, all cycles, in which variations of $\sigma_{min}$, $\sigma_{max}$, $\varepsilon_{min}$, $\varepsilon_{max}$ of more than 1 % with respect to the preceding cycle were detected.

Data within individual cycles were recorded using combined acquisition rates. Data were recorded closely during the ramps in all experiments, i.e., using small time increments. The time increment was increased during the dwell times to keep the total data volume reasonably low. However, additional data were recorded mainly at the beginning of the dwell times at smaller time steps to be able to analyze nonlinear stress or strain changes with sufficient accuracy. In summary, data were collected at the following time increments (TI):

RF:

• during ramps: TI = 0.08 s
• during dwell times: TI = 1.80 s
• additionally, during the first six seconds of the compressive dwell (DT_min_): TI = 0.08 s
• additionally, during the last two seconds of the compressive dwell (DT_min_): TI = 0.08 s
• additionally, during the first four seconds of the tensile dwell (DT_max_): TI = 0.08 s
• additionally, during the last two seconds of the tensile dwell (DT_max_): TI = 0.08 s

SLR and SLC:

• during ramps: TI = 0.08 s
• during dwell times: TI = 5.98 s
• additionally, during the first 10 seconds of the compressive dwell (DT_min_), the SSO-dwell and the tensile dwell (DT_max_): TI = 0.08 s
• additionally, during the first 60 seconds of the compressive dwell (DT_min_), the SSO-dwell and the tensile dwell (DT_max_) TI = 2.00 s

The data type “single” allowed recording the data at a time resolution of eight digits with variable decimal separator position.

## 3 Experimental Design, Materials and Methods

Experimental design: Three types of complex creep-fatigue experiments were conducted isothermally at 620°C in air on grade P92 steel with a mechanical strain amplitude of 0.2 % and a strain ratio of R = -1. They led to the sophisticated, test-specific stress-strain relations shown in Fig. 1:

(1) Strain-controlled relaxation fatigue (RF) test with strain-controlled three-minute dwell times (DT) at minimum and maximum strain (referred to as DT_min_ and DT_max_) in each cycle [1,2];
(2) service-like relaxation fatigue (SLR) with strain-controlled three-minute DT_min_ and DT_max_ and additional 30-minute dwells at constant strain (ε = 0, which represent steady-state operation (therefore designated as SSO(SLR) periods) [1], and
(3) service-like creep-fatigue (SLC) with strain-controlled three-minute DT_min_ and DT_max_ and additional 30-minute dwells at constant stress [σ(ε = 0)] (designated as SSO(SLC) periods) [1].

**Fig. 2:**
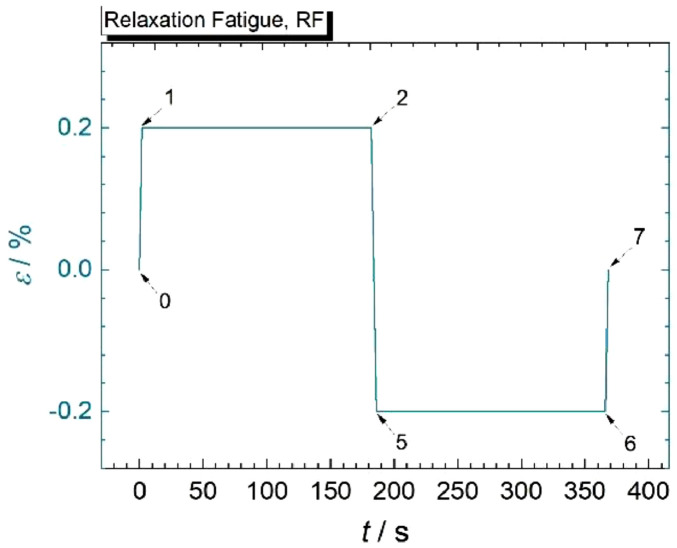
Loading path of fully strain-controlled relaxation fatigue (RF) test. Specific loading points (0 to 7) are numbered in analogy to those of the SL tests (Fig. 3) for the sake of comparability, i.e., points 3 and 4 are missing in this scheme due to the absence of an SSO period for this type of test [2]. Specific information on the depicted loading segments of one standard RF cycle are summarized in Table 1.

**Fig. 3:**
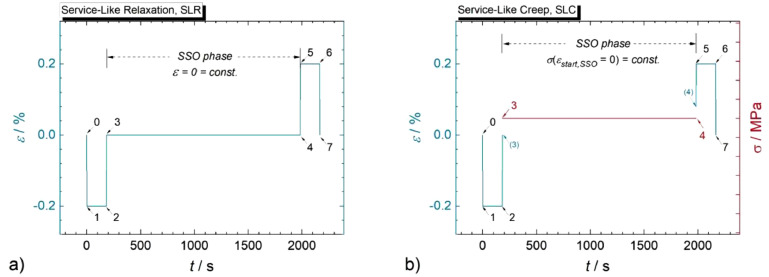
Loading paths of service-like (SL) tests with three-minute dwells at minimum and maximum strain and 30-min steady-state operation (SSO) phases in between for a) service-like relaxation (SLR) test with SSO at ε=0; b) service-like creep (SLC) test with SSO at stress values obtained at zero crossing of strain in each cycle [1]. Details of the depicted loading segments of the respective standard cycles (points 0 to 7) are summarized in Tables 2 and 3. (Figure reproduced from [1] without modifications under a CC BY 4.0 license).

The designs of experiments are illustrated in Fig. 2 for the RF test and in Fig. 3a,b for both SL tests, and specifications for all segments of the corresponding loading paths are listed in Table 1, Table 2, Table 3.

**Table 1:** Summary of RF test design. Description of loading segments according to the numbering in Fig. 2.

Relaxation Fatigue, RF					
Loading Segment	Control Mode	Action	Segment Duration/ s	Temperature /°C	Ref.
$\bar{01}$	Strain	*ramp* → ε_max_=0.2 %	2	620	[1,2]
$\bar{12}$	Strain	*DT_max_*: ε_max_=0.2%=const.	180		
$\bar{25}$	Strain	*ramp* → ε_min_=**-**0.2%	4		
$\bar{56}$	Strain	*DT_min_*: ε_min_=**-**0.2%=const.	180		
$\bar{67}$	Strain	*ramp* → ε=0	2

**Table 2:** Summary of SLR test design. Description of loading segments according to the numbering in Fig. 3a.

Service-Like Relaxation, SLR					
Loading Segment	Control Mode	Action	Segment Duration/ s	Temperature /°C	Ref.
$\bar{01}$	Strain	*ramp*→ε_min_=**-**0.2%	2	620	[1]
$\bar{12}$	Strain	*DT_min_*: ε_min_=**-**0.2%=const.	180		
$\bar{23}$	Strain	*ramp*→ε=0	2		
$\bar{34}$	Strain	*SSO(SLR)*: ε=0=const.	1800		
$\bar{45}$	Strain	*ramp*→ε_max_=0.2%	2		
$\bar{56}$	Strain	*DT_max_*: ε_max_=0.2%=const.	180		
$\bar{67}$	Strain	*ramp*→ε=0	2

**Table 3:** Summary of SLC test design. Description of loading segments according to the numbering in Fig. 3b.

Service-Like Creep, SLC					
Loading Segment	Control Mode	Action	Segment Duration/ s	Temperature /°C	Refs.
$\bar{01}$	Strain	*ramp*→ε_min_=**-**0.2%	2	620	[1]
$\bar{12}$	Strain	*DT_min_*: ε_min_=**-**0.2%=const.	180		
$\bar{23}$	Strain	*ramp*→ε=0	2		
$\bar{45}$	Load (Force)	*SSO(SLC)*: σ=const.	1800		
$\bar{45}$	Strain	*ramp*→ε_max_=0.2%	2		
$\bar{56}$	Strain	*DT_max_*: ε_max_=0.2%=const.	180		
$\bar{67}$	Strain	*ramp*→ε=0	2		

Material: The creep-fatigue tests were conducted for a grade P92 steel (X12CrMoWVNbN10-1) with a tempered martensite-ferritic microstructure and the chemical composition Fe–0.126C–0.114Si–0.446Mn-0.012P–0.005S–8.930Cr–0.167Ni–0.500Mo-0.019Co-0.092Nb-0.169V-1.95W (mass%). Cylindrical specimens of 18 mm gauge length and 6 mm gauge diameter were machined from a virgin pipe with a wall thickness of 47 mm in tangential direction. The specimens were tested with ground surface conditions (surface roughness, R_z_ ≤ 1). For a technical drawing of the specimen geometry and for the material's microstructure, it is referred to [2]. The original steam pipe was a fully heat treated section, provided by a commercial manufacturer (Vallourec, Germany). The well-controlled and reproducible industrial manufacturing process ensured representative material properties and high homogeneity of the material.

**Fig. 4:**
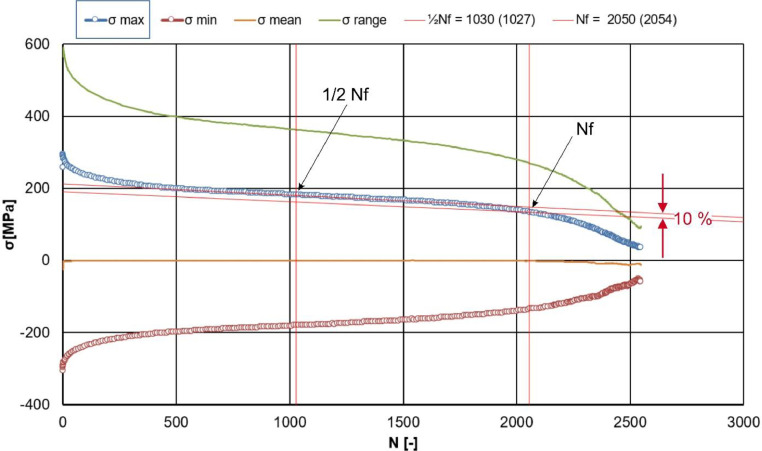
Peak stresses, $\sigma_{\text{min}}$, $\sigma_{\text{max}}$, vs. the number of fatigue cycles, N, exemplarily for the RF test. The number of cycles to failure, N_f_, was determined using the failure criterium of 10 % load drop of the maximum stress, $\sigma_{\text{max}}$, with respect to the slope of the stabilized deformation region (narrow parallel red lines). Plotted mean stresses and stress ranges can be determined from the “Max-min” stress data in the second sections of the respective *.lis files via $(\sigma_{\text{max}}+\sigma_{\text{min}})/2$ and $\sigma_{\text{max}}-\sigma_{\text{min}}$, respectively.

Methods: All tests were conducted in air, using a servo-hydraulic test machine with 100 kN force transducers (MTS Landmark, MTS, Eden Prairie, USA). Mechanical strains were measured by a water-cooled high-temperature extensometer (MTS-632.51F.04, MTS, Eden Prairie, USA) with an initial gauge length of 12 mm. All strain ramps (cf. Table 1, Table 2, Table 3) were performed at a constant strain rate of 1.0  ×  10^-3^ 1/s. Specimens were inductively heated to a target temperature of 620°C, which was indicated and controlled by a type S thermocouple of 0.2 mm diameter, spot-welded to the center of the gauge length. A plug-in high stability temperature controller (Eurotherm 2604, Schneider Electric Systems, Limburg, Germany) was used to measure and control the temperature. After fatigue testing, the number of cycles to failure, *N_f_*, was determined by applying the failure criterion of 10% load drop of the maximum peak stress against the stabilized cyclic deformation region, as illustrated in Fig. 4.

## Ethics Statements

The present work did not involve human subjects or animal experiments. No sensitive personal data was used.

## CRediT authorship contribution statement

**Nadja Sonntag:** Investigation, Formal analysis, Visualization, Writing – original draft, Writing – review & editing. **Maria Jürgens:** Investigation, Formal analysis. **Patrick Uhlemann:** Software, Data curation, Writing – original draft. **Birgit Skrotzki:** Writing – review & editing. **Jürgen Olbricht:** Conceptualization, Project administration, Funding acquisition, Writing – review & editing.

## Declaration of Competing Interest

The authors declare that they have no known competing financial interests or personal relationships that could have appeared to influence the work reported in this paper.

## Data Availability

Experimental Data from Service-Like Creep-Fatigue Experiments on Grade P92 Steel (Original data) (Zenodo).
